# Body size regulation by maturation steroid hormones: a *Drosophila* perspective

**DOI:** 10.1186/s12983-018-0290-9

**Published:** 2018-11-20

**Authors:** Seogang Hyun

**Affiliations:** Department of Life Science, Chung-Ang University, Heukseok-ro, Dongjak-gu, Seoul, 06974 Republic of Korea

**Keywords:** Body size, Steroid hormone, Ecdysone, Insulin/insulin-like growth factor/target of rapamycin signaling, Prothoracic gland, Fat body, Imaginal morphogenesis protein-late 2, *Drosophila*

## Abstract

The mechanism that determines the specific body size of an animal is a fundamental biological question that remains largely unanswered. This aspect is now beginning to be understood in insect models, particularly in *Drosophila melanogaster*, with studies highlighting the importance of nutrient-responsive growth signaling pathways involving insulin/insulin-like growth factor signaling (IIS) and target of rapamycin (TOR) (IIS/TOR). These pathways operate in animals, from insects to mammals, adjusting the growth rate in response to the nutritional condition of the organism. Organismal growth is closely coupled with the process of developmental maturation mediated by maturation steroid hormones, which is influenced greatly by environmental and nutritional conditions. Recent *Drosophila* studies have been revealing the mechanisms responsible for this phenomenon. In this review, I summarize some important findings about the steroid hormone regulation of *Drosophila* body growth, calling attention to the influence of developmental nutritional conditions on animal size determination.

## Introduction

How animals “know” when they should stop growth to achieve appropriate body size is one of the fundamental questions in biology. Body size impacts many aspects of an animal’s biology, from its physiology to its behavior. Misregulation of size control gives rise to multiple pathologies, from dwarfism and gigantism, to hypo- and hyperplasia of individual organs. The control of body size is therefore a key developmental process that ensures an animal grows to a body size that is optimal for its survival. Over the past few decades, studies from cell growth, cell proliferation, organ formation, and endocrine regulation have generated a long list of genes, signaling pathways and hormones that comprise the process of body size determination. Insects such as *Drosophila melanogaster* have been emerging as valuable models for the investigation of the process of animal size determination, enabling the integration of physiological studies with genetics. Insulin/insulin-like growth factor signaling (IIS)/target of rapamycin (TOR) (IIS/TOR) is highly conserved throughout the animal kingdom, integrating nutritional condition, energy metabolism, and cell proliferation. IIS/TOR signaling plays a major role in increasing larval body mass and postnatal body size in *Drosophila* and mammals, respectively, with an intimate connection to developmental maturation processes. Intriguing parallels between the regulation of body growth in insects and mammals have emerged, implicating surprisingly similar genes and signaling pathways. The mass of the mammalian body exponentially increases during the juvenile growth period, which has been proposed to correspond to the larval stage of the flies. As larvae prepare to mature by initiating puparium formation, the activity of the steroid hormone called ecdysone increases with the deceleration of body growth, thereby essentially finalizing the size of the adult body at the end of larval development. Similarly, mammalian body growth rapidly occurs from birth until puberty, followed by the influence of the peak activity of sex steroid hormones coinciding with the deceleration of body growth until adulthood. In this review, I will summarize some important findings from *Drosophila* studies that might elucidate the general principles underlying the regulation and determination of the appropriate size of the adult body.

## The nutrition-responsive growth pathway: IIS/TOR signaling

IIS/TOR pathway is a signaling cascade highly conserved across metazoan taxa. It acts as a nutrient-sensing pathway, transducing nutritional conditions to control cellular and organismal metabolism and growth. Studies over the past couple of decades have expanded our knowledge about this signaling pathway. Insulin and insulin-like peptides (ILPs) secreted from insulin-producing cells (IPCs) work in an endocrine manner by binding to insulin receptors (InRs) on the membrane of target cells in peripheral tissues, which triggers a phosphorylation cascade comprising insulin receptor substrate (IRS), phosphoinositide-3 kinase (PI3K), and Akt [1]. The PI3K complex consists of a catalytic subunit (p110) and a regulatory subunit (p85a) [2]. PI3K catalyzes the conversion of phosphatidylinositol 4,5-bisphosphate (PIP_2_) to phosphatidylinositol 3,4,5-triphosphate (PIP_3_) in the cell membrane [3], which is catalytically reversed by the activity of phosphatase and tensin homolog (PTEN) [4]. Accumulation of PIP_3_ in the cell membrane recruits PH domain-harboring proteins like phosphoinositide-dependent protein kinase 1 (PDK1) and Akt [1, 5]. The phosphorylation and activation of Akt by PDK1 subsequently cause the phosphorylation of various downstream effector proteins, one of which is the transcription factor Forkhead box O (FOXO). The phosphorylation of FOXO hinders its nuclear localization, thus suppressing its transcriptional activity [6]. The activity of IIS can be regulated in extracellular spaces by insulin-like growth factor (IGF)-binding proteins (IGFBPs). By binding to IGFs, IGFBPs not only prolong the half-lives of IGFs but also modulate their availability and activity [7].

A protein complex containing the TOR kinase (TORC1) is activated in a cell-autonomous manner either by signaling through insulin/PI3K/Akt or in response to the availability of extracellular nutrients. TOR can be activated by cell-autonomous sensing of nutrient availability, mediated by members of Rag GTPases [8]. Otherwise, activation of Akt by IIS stimulates the TOR pathway by suppressing the complex formed by tuberous sclerosis complex (TSC) 2, an inhibitor of TOR activity [9–12]. Recent studies have also found that Akt phosphorylates and represses PRAS40, another inhibitor of TOR, thereby enabling the IIS-induced activation of TOR [13, 14]. The activation of TOR kinase stimulates cell growth by increasing global protein translation with enhanced ribosome biogenesis, which is caused by the phosphorylation of the translation initiation factor 4E-binding protein (4EBP) and ribosomal protein S6 kinase (S6K), respectively [15].

An extensive crosstalk exists between IIS and TOR for elaborate control of growth and metabolism in response to fluctuating nutritional conditions. S6K activated by TOR phosphorylates IRS to inhibit IIS activity [16], which may prevent the overactivation of IIS by excessive nutrient stimuli. The activity of Akt can be promoted by TOR [17], which presumably ensures optimal metabolic homeostasis against harsh nutritional conditions. The expression of InR is induced by FOXO [18], a transcription factor repressed by Akt, which may potentiate the effects of ILPs on the IIS pathway upon starvation. For detailed information about the IIS/TOR pathways, the reader may refer to other excellent reviews [19, 20].

## Principles of body size determination in *Drosophila*

*Drosophila* are holometabolous insects; that is, they undergo complete metamorphosis during their life cycle. The life cycle consists of four distinct stages: egg, larva, pupa, and adult. The larva undergoes two molting steps, during which the mouth parts as well as the skins are shed. After the second molt, the larva (now third instar) feeds until ready to pupate. The progression of larval molting and pupal development is driven by pulses of the steroid hormone 20-hydroxyecdysone (20E) [21]. The prothoracic gland (PG), an insect endocrine organ, produces ecdysone, which is released into larval hemolymph and is converted into the active form 20E in peripheral tissues [22, 23]. 20E enters its target cells across lipid bilayers by simple diffusion or facilitated diffusion mediated by a membrane transporter [24]. Entered 20E then binds to a heterodimer nuclear receptor complex consisting of ecdysone receptor (EcR) and ultraspiracle (Usp), triggering stage-specific transcriptional cascades, and thus inducing the progression of waves of stages in fly development [21]. It is noted that although best known as molting hormone, ecdysone has also been known to affect other physiology such as reproduction, immunity, and lifespan [25–27].

Early in the third instar, the larva grows to reach a physiologically important developmental checkpoint, termed as critical weight (CW) [28–30]. Upon the attainment of the CW, the larva is believed to store enough nutrients within the body to complete the remaining larval development without further nutrient feeding. Whether or not the starvation of the developing larva delays the timing of puparium formation depends on the timing of starvation relative to the CW checkpoint [30]. When larvae are starved before reaching the CW, they pause the developmental progression until the normal nutritional condition is restored. However, when the larvae experience starvation past the CW, they nevertheless continue to develop into the pupal stage without developmental delay.

Final adult size is determined virtually by two developmental parameters during the larval growth period: the duration of the larval growth period and the rate of larval body growth (body volume increase per time). As briefly mentioned above, body size drastically increases during the larval period, contributing to most of the final body size. As such, a small-sized adult can be formed by two possible ways: (1) by shortening the duration of the larval growth period without modifying the rate of body growth or (2) by reducing the rate of body growth without modifying the duration of the larval growth period. It has been known that in *Drosophila*, malnutrition during larval development decreases the rate of body growth caused by a reduction in peripheral IIS. However, temporal malnutrition during larval development does not necessarily lead to a small-sized adult. When the starvation is temporally experienced before the CW is attained, the rate of body growth is reduced, but concomitant extension of the larval period compensates the lower growth rate to achieve normal body size. Meanwhile, starvation past the CW checkpoint decreases the rate of body growth without extending the duration of the larval period, which leads to a small-sized adult. This is consistent with the finding that transient inactivation of InR before the CW checkpoint lengthens the total larval period without affecting final body size, whereas InR inactivation after the CW checkpoint decreases the final body size without affecting total developmental time [31] (see below).

Although most of the body size is achieved during the larval period, a small fraction of size increase appears to occur after the cessation of larval feeding. The marginal growth during postfeeding larval and pupal stages has been shown to be mediated by *Drosophila* insulin-like peptide 6 (dILP6), which is secreted from the fat body in response to the pupariation signal and FOXO activity [32, 33]. dILP6 may mediate the tradeoff between body growth and storage of energy resources for pupal development, leading to appropriately sized flies with resistance to nutritional shortage.

## Control of larval body growth by the fat body, the nutrient-sensing organ

The fat body is the insect metabolic organ with functions similar to those of the mammalian liver and adipose tissues. It has been thought to have the ability to monitor the nutritional condition of the organism, storing or mobilizing energy resources in the form of glycogen and lipids. Accumulated evidence indicates that the fat body plays an endocrine role by producing certain hormonal peptides in the hemolymph, regulating the systemic metabolism and growth. A pioneering study by Leopold’s group showed that the inactivation of a cationic amino acid transporter specifically in the larval fat body suppresses the TOR signaling in this organ, causing the deceleration of organismal growth. [34]. They proposed that the secreted factor(s) emanating from the fat body diffuse into peripheral tissues and modulate their IIS, thereby controlling the rate of body growth.

Many humoral peptides that may have growth-modifying effects have been known to be expressed in the fat body. Acid-labile subunit (ALS) is a binding partner of IGF-1, which stabilizes and, simultaneously, restrains the activity of IGF-1 in mammals [35]. The *Drosophila* homolog of ALS (dALS) has been shown to be expressed in the larval fat body and form a complex with the *Drosophila* homolog of ILPs (dILPs) in a manner similar to that of its mammalian counterparts [36]. Imaginal morphogenesis protein-Late 2 (Imp-L2) is another binding partner of dILPs, consistent with its sequence homology to human IGFBP-7 [37, 38]. Imp-L2 can form a ternary complex with dILP2 and dALS, and it appears to block dILP activity [37, 38]. Consistently, the deletion of Imp-L2 leads to a bigger adult fly [37]. Neural Lazarillo (NLaz), the *Drosophila* lipocalin family member homologous to retinol-binding protein 4, has been shown to be the secreted protein that attenuates IIS [39]. NLaz mutant flies are bigger in size and exhibit an increase in peripheral IIS [39]. The mechanism by which secreted NLaz modulates peripheral IIS remains unknown.

Subsequent studies identified additional humoral factors from the fat body that modulate systemic IIS in different ways. By employing an ex vivo co-culture system for the fat body and brain tissues containing IPCs, Leopold’s group provided evidence supporting that dILP secretion from IPC is promoted by humoral factor(s) derived from the fat body [40]. Several humoral proteins that appear to mediate this effect have been characterized. Unpaired 2 (Upd2), a protein with sequence similarity to type 1 cytokine, was shown to be secreted from the fat body of well-fed larvae, activating JAK/STAT signaling in GABAergic neurons in the vicinity of IPCs. The activation of JAK/STAT signaling in these neurons then relieves their inhibitory effect on the IPC, thereby releasing dILPs into the hemolymph [41]. Eiger, the *Drosophila* homolog of TNF-α, is a proinflammatory cytokine involved in several aspects of IIS inhibition. It was found that upon low-protein diet, TNF-α-converting enzyme (TACE) is activated in the larval fat body, permitting the cleavage and release of active Eiger in the hemolymph. It acts on its receptor Grindelwald on IPCs, leading to JNK-dependent inhibition of dILP production [42]. Methuselah (Mth) is a G protein–coupled receptor (GPCR), mutations of which have been known to extend fly lifespan [43]. A recent study showed that Mth and its ligand Stunted (Sun) are expressed in larval IPCs and the fat body, respectively. Sun is secreted from the fat body in well-fed larvae, which is dependent on TOR activity in the fat body. The secreted Sun then acts on Mth on IPCs, thereby stimulating the release of dILPs from IPCs into the hemolymph [44]. CCHamid2 (CCHa2) peptide and its GPCR receptor CCHa2-R have also been shown to be expressed in the larval fat body and IPCs, respectively. CCHa2 is expressed in response to sugar and protein dietary sources, causing the production of dILPs from IPCs by acting on CCHa2-R [45]. Lastly, two growth-blocking peptides (GBPs), GBP1 and GBP2, have been shown to be expressed in the larval fat body in response to amino acids and TOR signaling. Secreted GBP1 and GBP2 from the fat body could stimulate dILP secretion from the IPCs, although the receptors or direct targets of the GBPs remain uncharacterized [46]. It is interesting to note that the expression of the humoral proteins affecting dILP production from IPCs appears to depend specifically on different macronutrients. Upd2 expression appears to be induced by fat and sugar rather than by amino acids, while Eiger, Sun, and GBP appear to respond to the availability of dietary proteins. The humoral proteins derived from the fat body described herein are summarized in Table 1.

**Table 1:** Summary of humoral proteins produced from the fat body described in the text

Humoral protein	Effects on body growth	Mechanism	Regulation in fat body
dALS	Suppress	Bind and antagonize dILP	Unknown
Imp-L2	Suppress	Bind and antagonize dILP	Upregulated by ecdysone
NLaz	Suppress	Unknown	Upregulated by JNK
Upd2	Enhance	Stimulate dILP production	Upregulated by sugar and fat diet
Eiger	Suppress	Inhibit dILP production	Downregulated by protein diet
Stunted	Enhance	Stimulate dILP production	Upregulated by protein diet
CCHamid2	Enhance	Stimulate dILP production	Upregulated by sugar and protein diet
GBP1, GBP2	Enhance	Stimulate dILP production	Upregulated by protein diet

**Fig. 1:**
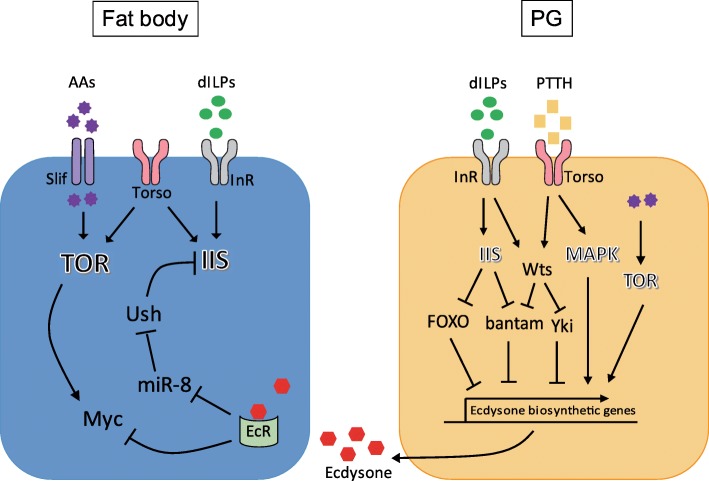
The outline of the molecular pathways in the fat body and prothoracic gland (PG) implicated in ecdysone-mediated body growth and size determination. In the fat body, amino acids (AAs) transported via the Slimfast (Slif) transporter stimulate the activity of target of rapamycin (TOR) signaling. *Drosophila* insulin-like peptides (dILPs) bind to and activate the insulin receptor (InR), thereby increasing the activity of the insulin/insulin-like growth factor signaling (IIS) pathway. Activation of the ecdysone receptor (EcR) by ecdysone binding inhibits the expression of miR-8, thereby activating the expression of U-shaped (Ush), an inhibitor of IIS. Activated EcR also represses Myc, activated by TOR signaling. Through these pathways, ecdysone suppresses IIS/TOR activity, thereby attenuating the rate of body growth. In the PG, activated IIS suppresses bantam, a repressor of the expression of ecdysone biosynthetic genes. FOXO, which is suppressed by IIS, binds to ultraspiracle (Usp) thereby inhibiting the expression ecdysone biosynthetic genes. PTTH peptides released from the axon terminals of PTTH-expressing neurons acts on Torso, the PTTH receptor, which activates MAPK signaling cascades, thereby activating ecdysone generation. Activated InR or Torso also stimulates Warts (Wts), which in turn represses the activity of bantam and Yorki (Yki), thereby controlling ecdysone synthesis. TOR signaling activated by a protein-rich diet promotes ecdysone generation. Torso is also expressed in the fat body, stimulating IIS/TOR. The ligands acting on Torso in the fat body are currently unknown

The ability of the fat body to coordinate organismal metabolism and growth in response to systemic nutritional condition via the endocrine pathways described above leads to the theory that IIS/TOR in the fat body plays a central role in larval body growth and adult size determination. Although it is less clear whether IIS and TOR signaling act in parallel or in an inter-connected pathway in the larval fat body, the suppression of either one in the fat body can cause a decrease in body size. Inactivation of an amino acid transporter, Slimfast (Slif), in the fat body suppresses the fat body’s TOR activity, giving rise to a small-sized adult [34]. The suppression of IIS in the fat body by the inactivation of InR or PI3K attenuates organismal growth [47, 48], and activation of Akt or InR in the fat body rescues the small body size induced by immune responses or knockdown of Torso, a receptor tyrosine kinase, respectively [49, 50] (see below). These findings have highlighted the fat body as the central organ mediating nutrition-dependent growth of the developing animal (Fig. 1).

## Effects of ecdysone on the control of body size

The attainment of the CW checkpoint appears to coincide with the onset of three serial low-titer pulses of 20E in the early 3rd instar larval stage [31, 51]. This implies that the PG may play a critical role in determining final body size by regulating the attainment of CW and the duration of the larval growth period. The suppression of ecdysone production by the inactivation of PI3K, Ras, or Raf specifically in the PG leads to increased final adult size by extending the larval growth period without affecting the rate of body growth [28, 52]. Interestingly, it was found that ecdysone could affect final body size not only by altering the duration of the larval growth period but also by changing the rate of body growth through the modulation of peripheral IIS. Modulating PI3K in the PG in some cases modifies final body size without affecting the timing of pupariation [53]. Preventing EcR signaling in peripheral tissues increases the final adult size, stimulates the activities of PI3K and Akt, and blocks the nuclear localization of FOXO. These observations suggest that ecdysone not only induces developmental transitions such as molting and puparium formation but also attenuates IIS activity in peripheral tissues during the larval growth period [53]. A subsequent study found that ecdysone mainly acts on EcR in the cells of the larval fat body, which suppresses the activity of the Myc transcription factor in the fat body and the subsequent repression of peripheral IIS and body growth [54].

microRNAs (miRs) participate in the ecdysone-dependent growth of the fly. miR-8 is a highly conserved miR across metazoan species, and it has been found to promote IIS in both flies and humans by suppressing a common target gene, *u-shaped* (*ush*) [47]. The human homolog of Ush was shown to bind to and block the activity of PI3K and thereby inhibit IIS [47]. Numerous binding sites for the proteins encoded by ecdysone’s early response genes were found in the promoter region of miR-8, rendering it transcriptionally repressed by ecdysone signaling. Increased concentration of circulating ecdysone in the hemolymph was proposed to suppress miR-8 expression, which increases Ush expression and the subsequent attenuation of peripheral IIS and body growth, probably via fat body-derived hormones [55, 56]. bantam is an insect-specific miR well-known for its cell-autonomous mitogenic effects in imaginal epithelial tissues in developing *Drosophila* larva [57, 58]. Interestingly, bantam can also regulate systemic body growth in a non cell-autonomous manner. The expression of bantam in the PG suppresses the biogenesis of ecdysone, which reduces the basal ecdysone level in the hemolymph, thereby increasing peripheral IIS activity and the rate of larval body growth. In turn, the increase in IIS in the PG suppresses the activity of bantam, thereby connecting peripheral IIS and ecdysone production [59]. A recent study further showed that Warts (Wts) signaling mediates the ecdysone-synthesizing effects of dILPs and prothoracicotropic hormone (PTTH), a neuropeptide that activates ecdysone biogenesis via its receptor Torso. The author found that Wts downregulates the activity of bantam and Yorki (Yki), thereby controlling ecdysone synthesis and larval growth [60]. The gene targets of bantam that are responsible for the control of ecdysone generation remain to be identified.

Intriguingly, Torso was also found to be expressed in the fat body, accelerating the rate of larval body growth. Torso was shown to activate IIS/TOR, probably by acting as a receptor tyrosine kinase [50]. Although the ligand that acts on Torso in the fat body is yet to be identified, PTTH could be a candidate ligand based on the recent finding that as well as a neuropeptide secreted from axonal terminals, PTTH can also function as a hormone, acting on distantly located organs [61]. If this is the case, it could be postulated that PTTH could differently affect fly body growth either by stimulating ecdysone production from PG or by activating IIS/TOR in the fat body.

*Drosophila* imaginal discs are larval tissues that give rise to the adult appendage after undergoing metamorphosis. When the growth of the imaginal disc is perturbed, the duration of the larval growth period is prolonged by the postponement of pupariation [62–67]. It has been speculated that damaged imaginal discs may emit some humoral signals that affect the biogenesis of ecdysone in the PG, thereby communicating local growth perturbation to the center of developmental timing. Recent studies have identified the humoral proteins that may mediate this communication. dILP8, named for its invariant 6-cysteine motif typical of dILPs, is produced in imaginal disc tissues exhibiting perturbed growth, which causes delayed pupariation [68, 69]. The depletion of dILP8 abrogates the developmental delay caused by local growth perturbation, while ectopic overexpression of dILP8 decreases the expression of ecdysone biosynthetic genes and delays the timing of pupariation. The orphan relaxin receptor Lgr3 was recently characterized as the receptor for dILP8. Lgr3-expressing neurons appear to physically associate with the neurons expressing PTTH. This neuronal architecture may allow dILP8’s modulation of ecdysone production from the PG [70–72]. Together, these findings suggest that local organ growth, as well as organismal growth, is tightly connected with the developmental maturation process.

Although not being a steroid, the juvenile hormone (JH) has been known to control the timing of the metamorphic transition and hence growth duration, together with ecdysone. Increasing evidence indicates that JH also participates in regulating final insect size. In the tobacco hornworm *Manduca sexta*, a decline in circulating JH titer initiates the hormonal cascade that begins with attainment of CW, followed by the rise of circulating ecdysone that finally stops body growth [30, 73, 74]. The role of JH in the regulation of *Drosophila* body size and IIS was recently investigated. The larvae lacking the corpora allata (CA), the JH-producing organ, was shown to pupariate at smaller sizes than control larvae due to a reduced larval growth rate. Notably, these mutant larvae exhibited decreased IIS activity accompanied by elevated ecdysone signaling, implying that JH could regulate body growth and final adult size by antagonizing ecdysone activity [75].

## Nutritional control of *Drosophila* body size mediated by ecdysone

The nutritional condition during the larval growth period can have a huge impact on the size of the adult fly by affecting the CW or the rate of larval body growth, which could be mediated by IIS/TOR in the PG or in the fat body, respectively. As mentioned above, the inhibition of the activity of PI3K, a component of IIS, in the PG suppresses the biogenesis of ecdysone, which leads to increased size of the adult fly either by lengthening the larval growth period or by accelerating the rate of larval body growth [28, 52, 53]. The effect of PI3K on the biogenesis of ecdysone led to further investigation of the effect of IIS/TOR on ecysone production. It was shown that decreasing TOR signaling in the PG delays the timing of pupariation by modulating the biogenesis of ecdysone [76], and a recent study further showed that conversion of ecdysone to its active form 20E by the enzyme Shade is nutritionally regulated in the fat body [77]. These studies suggest a mechanism for nutritional control of ecdysone production and activity, which together with the effects of ecdysone on IIS in peripheral tissues described above, constitutes the organismal feedback loop between ecdysone and IIS (Fig. 1).

A recent study from Mirth’s group showed that starvation occurring in larvae prior to reaching the CW decreases circulating ecdysone levels, thereby delaying the time to reach the CW and pupariation [78]. The authors proposed a model according to which the reception of the nutritional condition of the organism by the PG via IIS/TOR influences the interaction of FOXO with Usp in the nucleus in PG cells, which affects Usp-mediated transcription of ecdysone biosynthetic genes [78]. Additional mechanism was recently suggested by the study showing that TOR activation in the PG facilitates endoreplication in the PG cells, which is required for efficient synthesis of ecdysone to progress over CW checkpoint [79]. In these ways, in the early larval stage before third instar, poor nutrition appears to limit ecdysone production from the PG, thereby suppressing the CW.

Interestingly, my group recently found that after the CW is attained, malnutrition in the third instar larvae actually increases ecdysone levels in the hemolymph. This causes ecdysone signaling-dependent Imp-L2 production from the fat body into the hemolymph, as evidenced by the observation that the inactivation of EcR blocks the expression of Imp-2 induced by starvation. Because Imp-L2 in the hemolymph suppresses peripheral IIS and body growth, a working model has been proposed as follows: (1) poor nutrition in third instar larvae past the CW stimulates ecdysone production in the PG, thus elevating the basal levels of circulating ecdysone; (2) this increases the activity of ecdysone signaling in the fat body, stimulating Imp-L2 production from the fat body; and (3) finally, elevated hemolymph Imp-L2 suppresses peripheral IIS and body growth, eventually leading to smaller sized adults [80].

**Fig. 2:**
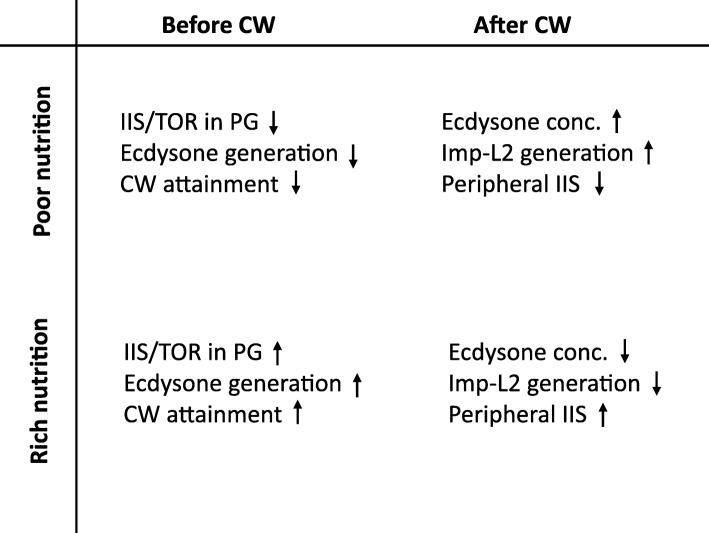
Summary of the changes in key physiological parameters involved in ecdysone-mediated growth in response to each combination of different developmental and nutritional conditions. Detailed information is described in the text

Unexpectedly, Imp-L2 expression in the fat body upon starvation still occurs, regardless of the inactivation of IIS/TOR in the fat body. This is surprising, since the fat body IIS/TOR has long been believed to be the control center for nutrition-dependent organismal growth, as described in the previous section. Furthermore, in contrast with the phenotype resistant to nutrition restriction, *Imp-L2*-null mutants exhibited a rapid decrease in body size in response to the inhibition of amino acid transport into the fat body. These findings suggest that nutritional control of body growth may use multiple parallel endocrine pathways, which might involve additional nutrient-sensing mechanisms besides IIS/TOR in the fat body. Accordingly, the nutritional information processed by some yet undetermined nutrient-sensing mechanism could be delivered to peripheral tissues via the ecdysone–fat body–Imp-L2 pathway [80]. Together, these studies suggest that the nutritional condition during larval development may differentially regulate the size of the adult body, depending on the timing of nutritional stress with respect to a CW developmental checkpoint at the third instar larval stage (Fig. 2).

## Conclusions

The life history of animals consists of discrete stages, such as embryonic development, juvenile growth, sexual maturation, and reproductive adulthood. Among the many environmental and genetic cues, nutrient availability is thought to have major effects on juvenile body growth and final adult size. Juvenile body growth is closely linked with the process of developmental maturation mediated by maturation steroid hormones, which is hugely affected by environmental and nutritional conditions. Numerous studies in diverse animal species have been highlighting IIS/TOR as the main effector pathway in driving developmental growth and maturation depending on the nutritional status of the organism. Since long, *Drosophila* has been serving as the most valuable model for understanding the genetic and endocrine pathways that coordinate juvenile growth and maturation, generating fascinating theories that may help establish general principles underlying size determination of adult animals. The IIS/TOR in the fat body serves as internal nutrient sensor that orchestrates the nutritional condition-dependent growth of the *Drosophila* larva, a notion that has been recently complicated by findings of IIS/TOR-independent regulation of Imp-L2 and its involvement in hitherto unrecognized endocrine pathways. Early in the third instar, the larva grows to reach the CW developmental checkpoint, which appears to segregate the developmental phases when the nutritional condition differentially influences the rate of development and growth as follows. (1) Before the CW checkpoint is reached, poor nutrition limits ecdysone production from the PG, thereby suppressing CW and delaying pupariation timing. (2) After the CW checkpoint is crossed, poor nutrition stimulates ecdysone production from the PG, thereby attenuating peripheral IIS and the rate of body growth without delaying pupariation timing. On the basis of the similarities of the processes of developmental growth between flies and humans, future discoveries of these developmental mechanisms might help define a conserved genetic architecture regulating juvenile growth and maturation throughout the animal kingdom and provide a new direction for understanding how human puberty and growth are coordinately regulated.

## Abbreviations

20E: 20-hydroxyecdysone
AAs: Amino acids
CA: Corpora allata
CW: Critical weight
dALS: *Drosophila* homolog of Acid-labile subunit
dILP: *Drosophila* homolog of insulin like peptide
EcR: Ecdysone receptor
GBP: Growth-blocking peptide
IGFBP: Insulin like growth factor binding protein
IIS: Insulin/insulin like growth factor signaling
Imp-L2: Imaginal morphogenesis protein-Late 2
InR: Insulin receptor
JH: Juvenile hormone
NLaz: Neural Lazarillo
PG: Prothoracic gland
PTTH: Prothoracicotropic hormone
Slif: Slimfast
TOR: Target of rapamycin
Upd2: Unpaired 2
Ush: U-shaped
Usp: Ultraspiracle
Wts: Warts
Yki: Yorki
